# Organizational readiness for change towards implementing a sepsis survivor hospital to home transition-in-care protocol

**DOI:** 10.3389/frhs.2024.1436375

**Published:** 2024-09-06

**Authors:** Elaine Sang, Ryan Quinn, Michael A. Stawnychy, Jiyoun Song, Karen B. Hirschman, Sang Bin You, Katherine S. Pitcher, Nancy A. Hodgson, Patrik Garren, Melissa O'Connor, Sungho Oh, Kathryn H. Bowles

**Affiliations:** ^1^ NewCourtland Center for Transitions and Health, School of Nursing, University of Pennsylvania, Philadelphia, PA, United States; ^2^ Leonard Davis Institute of Health Economics, The Wharton School, University of Pennsylvania, Philadelphia, PA, United States; ^3^ Biostatistics Evaluation Collaboration Consultation Analysis (BECCA) Lab, School of Nursing, University of Pennsylvania, Philadelphia, PA, United States; ^4^ Department of Biobehavioral Health Sciences, School of Nursing, University of Pennsylvania, Philadelphia, PA, United States; ^5^ Penn Medicine Princeton Medical Center, Plainsboro Township, NJ, United States; ^6^ Gerontology Interest Group, M. Louise Fitzpatrick College of Nursing, Villanova University, Villanova, PA, United States; ^7^ Center for Home Care Policy & Research, VNS Health, New York, NY, United States

**Keywords:** sepsis survivors, transitions in care, organizational readiness for change, implementation science, healthcare system, home health care (HHC), transition-in-care protocols, hospital to home

## Abstract

**Background:**

Organizational readiness for change, defined as the collective preparedness of organization members to enact changes, remains understudied in implementing sepsis survivor transition-in-care protocols. Effective implementation relies on collaboration between hospital and post-acute care informants, including those who are leaders and staff. Therefore, our cross-sectional study compared organizational readiness for change among hospital and post-acute care informants.

**Methods:**

We invited informants from 16 hospitals and five affiliated HHC agencies involved in implementing a sepsis survivor transition-in-care protocol to complete a pre-implementation survey, where organizational readiness for change was measured via the Organizational Readiness to Implement Change (ORIC) scale (range 12–60). We also collected their demographic and job area information. Mann-Whitney *U*-tests and linear regressions, adjusting for leadership status, were used to compare organizational readiness of change between hospital and post-acute care informants.

**Results:**

Eighty-four informants, 51 from hospitals and 33 from post-acute care, completed the survey. Hospital and post-acute care informants had a median ORIC score of 52 and 57 respectively. Post-acute care informants had a mean 4.39-unit higher ORIC score compared to hospital informants (*p *= 0.03).

**Conclusions:**

Post-acute care informants had higher organizational readiness of change than hospital informants, potentially attributed to differences in health policies, expertise, organizational structure, and priorities. These findings and potential inferences may inform sepsis survivor transition-in-care protocol implementation. Future research should confirm, expand, and examine underlying factors related to these findings with a larger and more diverse sample. Additional studies may assess the predictive validity of ORIC towards implementation success.

## 1 Background

Older adults are vulnerable to poor health outcomes while transitioning from hospital to post-acute care, including home health care (HHC), outpatient appointments, rehabilitation care, and skilled or long-term care (1–4). This may be due to incomplete information transfer, medication reconciliation errors, and poor communication between and within transferring and receiving healthcare institutions (1–4). Transition-in-care protocols, defined as evidence-based guidelines and interventions intended to facilitate smooth care continuity as patients move between different healthcare institutions or stages of care (1–4), are critical to achieving positive patient outcomes, including fewer rehospitalizations, lower mortality, and better quality of life (4–8). Sepsis survivors, an at-risk population for high long-term morbidity and mortality, may especially benefit from transition-in-care protocols as they are nearly twice as likely to be rehospitalized within 30 days compared to the general patient population (18%–26% vs. 13.9%) within the United States (U.S.) (9, 10). Approximately 40% of sepsis survivor rehospitalizations in the U.S. are due to ambulatory care sensitive conditions (11). This suggests that they could have been prevented with early and intensive post-discharge follow-up, which may be facilitated by transition-in-care protocols (11).

Examples of transition-in-care protocols include the Transitional Care Model (1, 12–16), Care Transitions Intervention (1, 17, 18), and Better Outcomes for Older Adults Through Safe Transitions (1, 19). These protocols have been used for patients with advanced age, heart failure, depression, cognitive impairment, and multimorbidity (13–16, 18, 19). Key components of these protocols include discharge planning, nurse or physician post-discharge follow-up, and patient education (1, 4, 12–19). One transition-in-care protocol specific to sepsis survivors transitioning from hospital to home is I-TRANSFER (Improving TRansitions ANd outcomeS oF sEpsis suRvivors) and consists of an initial HHC nursing visit within two days, a second HHC nursing visit within seven days, and an outpatient appointment within seven days after hospital discharge (20). A previous comparative effectiveness national study using Medicare claims data from 170,571 sepsis survivors discharged from hospitals to HHC informed this protocol (21). This study found that sepsis survivors who received the I-TRANSFER protocol had a 41% relative reduction in 30-day rehospitalizations compared to those who did not (21). However, only 28% of sepsis survivors in the study received this type of care (21).

Transition-in-care protocols are often implemented by informants, or staff and leaders, within transferring (hospital) and receiving (post-acute care) healthcare institutions (6). Staff are those who execute tasks, follow protocols, or provide direct patient care. They mainly consist of clinicians and administrators. Clinicians include physicians, nurses, advance practice providers, physical and occupational therapists, social workers, and case managers. Examples of administrators include quality improvement professionals and information technology specialists. Leaders are those who are designated, based on their role, to instill motivation, build morale, create shared visions, and manage changes throughout the healthcare institution (22). They may include chief officers, directors, and upper-level management.

Successful protocol implementation is influenced by various individual, organizational, and external factors (4, 23). Individual factors include the role, motivation, expertise, and skills of the staff and leaders involved in the implementation (4, 23). Organizational factors may include staffing ratios, resource allocation, organizational priorities, and established internal workflows (4, 23). These workflows are systematic internal steps and processes followed by the transferring and receiving healthcare institutions when discharging or accepting patients. External factors may include health policies and quality initiatives from professional health organizations or government agencies (4, 23). An example of a government agency is the U.S. Centers for Medicare and Medicaid Services (CMS), which provides oversight on healthcare quality and safety provided by U.S. healthcare institutions (24).

Another important but unexplored factor within the context of implementing transition-in-care protocols is the organizational readiness for change among informants across the transferring (hospital) and receiving (post-acute care) healthcare institutions. Organizational readiness for change, defined by Weiner's theory on readiness for change, is the collective capacity of organizational members to psychologically and behaviorally embrace and implement organizational changes (25). High organizational readiness for change suggests greater collective capability and efficacy among organizational members towards implementing and adapting to changes, leading to more persistence and cooperation in adopting new initiatives (26). Meanwhile, low readiness for change contributes to resisting and viewing change as undesirable, leading to unsuccessful implementation efforts (26). To our knowledge, previous studies on organizational readiness for change focused on a single healthcare institution type, such as hospitals (23, 27, 28), but not across different types.

Effective implementation of transition-in-care protocols requires interdisciplinary collaborations between informants from hospital and post-acute care, both of which may have different organizational structures, care expertise, priorities, and relevant health policies. These factors may contribute to differences in organizational readiness for change. Understanding these differences may inform tailored strategies and health policies for implementing transition-in-care protocols, leading to better interdisciplinary collaborations and better patient outcomes.

Given the understudied nature of organizational readiness for change in transition-in-care protocol implementation and the potential benefits these protocols offer sepsis survivors, our study (1) described and compared informant characteristics based on leadership status and healthcare institution, and (2) determined whether healthcare institution is a predictor of organizational readiness for change. In this study we focused on the post-acute care institutions of outpatient clinics and HHC agencies.

## 2 Materials and methods

### 2.1 Overview and participants

This cross-sectional study was part of a larger type 1 hybrid effectiveness implementation science study that aims to measure the effectiveness and implementation of the I-TRANSFER protocol (20). Five healthcare systems (consisting of hospitals and outpatient clinics) and their five affiliated HHC agencies were purposively selected for implementing this protocol (20). They are diverse in size and geographic region (18). Three HHC agencies were owned by their affiliated healthcare system while the other two were free-standing (20). Some healthcare systems consisted of multiple hospitals. Specifically, one healthcare system had one hospital, another had two, another had three, another had four, and another had six, making a total of 16 hospitals involved in implementing the I-TRANSFER protocol. The larger study was approved by the Institutional Review Board of the University of Pennsylvania and VNS Health. Other healthcare systems and HHC agencies reviewed the protocol and granted permission after determining there was “no research engagement” of their patients or by their employees.

Informants eligible for our smaller cross-sectional study include staff and leaders from the five healthcare systems and HHC agencies. These informants were directly involved in implementing the I-TRANSFER protocol. They were recruited from our larger implementation science study. Findings from our smaller cross-sectional study are reported following the Strengthening the Reporting of Observational Studies in Epidemiology (STROBE) guidelines (Supplementary File 1) (29).

### 2.2 Data collection

Those eligible were invited and completed a pre-implementation organizational readiness for change survey via REDCap® (30, 31). This survey included questions on demographics (specifically gender, race, and ethnicity), job area (via 27 options), and healthcare institution (healthcare system or HHC). Example of job areas include outpatient staff, acute care director, and HHC case manager. They were allowed to select multiple job area options or type one in if no options applied. The survey was available from March 22, 2021 to October 15, 2021.

Organizational readiness for change was measured using the Organizational Readiness for Implementing Change (ORIC) scale (Supplementary File 2), which was developed by Shea et al. (26) and based on Weiner's theory on readiness for change. Although originally in English (26), the scale has been translated and psychometrically validated in multiple languages, such as German, Danish, French, and Brazilian Portuguese (32–35). ORIC contains 12 items, each rated on a five-point scale ranging from one (disagree) to five (agree) and are summed to generate an ORIC score ranging from 12 to 60 (26). This scale also includes two subscales: change commitment (five items) and change efficacy (seven items) (26). Change commitment refers to organizational members’ capability to implement change, while change efficacy represents their confidence and motivation to do so (26, 36). Subscale scores are calculated similarly to the ORIC score, with change commitment scores ranging from five to 25 and change efficacy scores from seven to 35 (26). Higher ORIC and subscale scores indicate higher organizational readiness for change, change commitment, and change efficacy (26).

To prepare for data analysis, job area was delineated by leadership status and was categorized into leader or staff. Healthcare institution was also operationalized as a dichotomous variable, indicating whether participants worked in either hospitals or post-acute care. Informants employed in healthcare systems and had job areas focused on coordinating outpatient appointments were categorized under post-acute care.

### 2.3 Data analysis

Cronbach's alpha was measured to assess the internal consistency of ORIC, change commitment, and change efficacy among informants. To describe the informant sample, frequencies and percentages were calculated for categorical measures (i.e., demographics, healthcare institution, and leadership status). Mean, standard deviation (SD), median, and interquartile range (IQR) were calculated for ORIC scores.

Box plots were used to illustrate the distribution of ORIC, change commitment, and change efficacy scores among informants in hospitals and post-acute care. Frequency bar graphs were also made to show informant responses to the 12 individual ORIC items. Responses were then separated based on healthcare institution (hospital vs. post-acute care). Stratified summary tables were produced to compare informants’ characteristics between healthcare institution as well as between leadership status.

Pearson's Chi-Square and Fisher's exact tests were used to test associations between categorical measures. The non-parametric Mann-Whitney *U*-test was used to test bivariate associations between ORIC score and the dichotomous measures of healthcare institution and leadership status. Finally, linear regression was used to assess the extent to which healthcare institution is predictive of ORIC score. We also adjusted for leadership status for there were significantly more informants who were leaders in post-acute care than in hospitals.

An alpha level of 0.05 was used to determine statistical significance. Data analysis was done using R version 4.2.2 (37). The package “ggplot2” was used create the box plot and bar graphs (38).

## 3 Results

Out of the 122 eligible informants who received the survey, 84 (68.85%) completed it and were included in the analysis. This included 51 (60.71%) from hospitals and 33 (39.29%) from post-acute care. Most informants were female (59/84, 70.24%), white (69/84, 82.14%), and non-Hispanic/non-Latino (78/84, 92.86%). Among those from post-acute care, four (12.12%) were from outpatient clinics and 29 (87.88%) were from HHC agencies. The sample was almost evenly split between informants in leadership (44/84, 52.38%) and staff roles (40/84, 47.62%). The mean score was 52.44 (SD 8.05) for ORIC, 22.15 (SD 3.45) for change commitment, and 30.28 (SD 4.93) for change efficacy. Supplementary File 3 describes the characteristics of the 84 informants.

Although hospitals and post-acute care both had 22 informants who were leaders, the proportion of leaders was higher in post-acute care (22/33, 66.67%) compared to hospitals (22/51, 35.48%) (*p* = 0.04). Neither healthcare institution nor leadership status were significantly associated with gender, race, or ethnicity (*p* > 0.05). However, leadership status was significantly associated with ORIC, change efficacy, and change commitment scores (*p* < 0.04). Table 1 stratifies these characteristics by healthcare institution and leadership status. Supplementary File 4 contains the frequency bar graphs showing informant responses to the 12 individual ORIC items, and Supplementary File 5 separated these responses by healthcare institution (hospitals and post-acute care).

**Table 1:** Characteristics based on healthcare institution and leadership Status of informants.

Characteristics	Hospital	Post-acute care	*P*-value	Leader	Staff	*P*-value
	(*n* = 51) *n* (%)	(*n* = 33) *n* (%)		(*n* = 44) *n* (%)	(*n* = 40) *n* (%)	
Gender			0.7			0.3
Male	16 (31.37%)	9 (27.27%)		11 (25.00%)	14 (35.00%)	
Female	35 (68.63%)	24 (72.73%)		33 (75.00%)	26 (65.00%)	
Race			0.8			0.2
White	42 (89.36%)	27 (84.38%)		39 (92.86%)	30 (78.95%)	
Asian	4 (8.51%)	5 (15.63%)		3 (7.14%)	6 (15.79%)	
Black	1 (2.13%)	0 (0.00%)		0 (0.00%)	1 (2.63%)	
Pacific Islander	1 (2.13%)	0 (0.00%)		0 (0.00%)	1 (2.63%)	
Ethnicity			0.5			>0.9
Hispanic/Latino	2 (4.17%)	0 (0.00%)		1 (2.50%)	1 (2.50%)	
Non-Hispanic/Non-Latino	46 (95.83%)	32 (100%)		39 (97.50%)	39 (97.50%)	
Leadership Status			0.04*	N/A	N/A	N/A
Leader	22 (43.14%)	22 (66.67%)				
Staff	29 (56.86%)	11 (35.48%)				
Healthcare Institution	N/A	N/A	N/A			0.04*
Hospital				22 (50.00%)	29 (72.50%)	
Post-Acute Care				22 (50.00%)	11 (27.50%)	

Organizational Readiness for Change	Median (IQR)	Median (IQR)	*P*-value	Median (IQR)	Median (IQR)	*P*-value
ORIC	52.50	57.00	0.03*	56.00	54.00	0.4
	(45.50, 59.00)	(53.50, 60.00)		(51.25, 59.75)	(46.25, 59.75)	
Change Commitment	23.00	24.00	0.02*	24.00	23.00	0.4
	(18.75, 25.00)	(22.75, 25.00)		(20.50, 25.00)	(20.00, 25.00)	
Change Efficacy	31.00	33.00	0.04*	32.41	30.50	0.4
	(24.75, 35.00)	(29.75, 35.00)		(29.25, 35.00)	(26.25, 35.00)	

* Indicates statistical significance.

**Figure 1:**
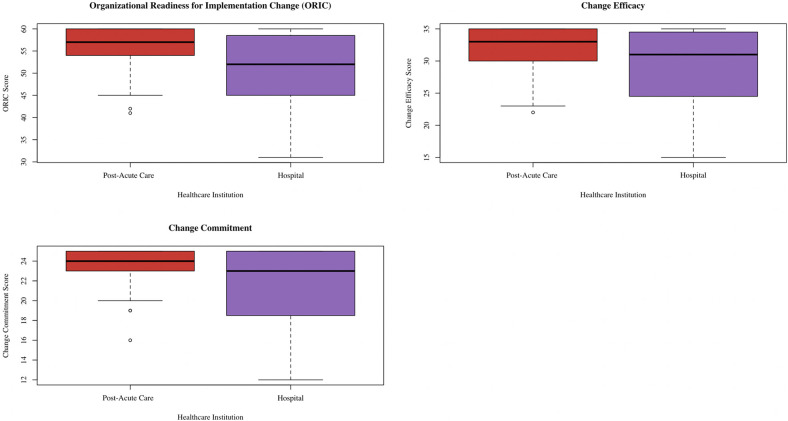
Box plot showing distribution of ORIC, change commitment, and change efficacy scores separated by healthcare institution. This box plot was created via the ggplot2 package (38) in R (37).

The median ORIC score was 52.50 (IQR 45.50, 59.00) among hospital informants compared to 57.00 (IQR 53.50, 60.00) among post-acute informants (*p* = 0.03) (26). For change commitment, the median score was 23.00 (IQR 18.75, 25.00) among hospital informants and 24.00 (IQR 22.75, 25.00) among post-acute informants (*p* = 0.02). Meanwhile, the median change efficacy score was 31.00 (IQR 24.75, 35.00) among hospital informants and 33.00 (IQR 29.75, 35.00) among post-acute informants (*p* = 0.04). There were no significant score differences based on leadership status. Figure 1 contains the box plots showing the ORIC, change commitment, and change efficacy score distributions, separated by healthcare institution. Cronbach's alpha was 0.96 for ORIC, 0.93 for change commitment, and 0.92 for change self-efficacy.

A simple linear regression model showed that post-acute informants had higher estimated mean ORIC (4.57 ± 1.74 units, *p* = 0.01), change commitment (1.94 ± 0.75 units, *p* = 0.01), and change efficacy (2.63 ± 1.07, *p* = 0.02) scores compared to hospital informants (Supplementary File 6). After adjusting for leadership status, post-acute care remained a significant predictor of ORIC (B = 4.39 ± 1.80 units, *p* = 0.02), change commitment (B = 1.93 ± 0.78 units, *p* = 0.01), and change efficacy (B = 2.46 ± 1.10 units, *p* = .03) (Table 2). Leadership status was not significantly associated with ORIC, change commitment, or change efficacy.

**Table 2:** Multiple linear regression of ORIC after adjusting for leadership Status.

Variables	Regression coefficient	Standard error	95% confidence interval	*t* value	*P*-value
ORIC					
Intercept	51.07	1.48	48.12, 54.02	34.45	<0.01*
Healthcare institution					
Hospitals	Reference				
Post-acute care	4.39	1.80	0.81, 7.96	2.44	0.02*
Leadership status					
Leader	Reference				
Staff	−0.76	1.76	−4.25, 2.74	−0.43	0.67
Change commitment					
Intercept	21.42	0.64	20.16, 22.69	33.67	<0.01*
Healthcare institution					
Hospitals	Reference				
Post-acute care	1.93	0.78	0.39, 3.46	2.50	0.01*
Leadership status					
Leader	Reference				
Staff	−0.05	0.75	−1.55, 1.45	−0.07	0.95
Change efficacy					
Intercept	29.65	0.91	27.84, 31.46	32.59	<0.01*
Healthcare institution					
Hospitals	Reference				
Post-acute care	2.46	1.10	0.27, 4.66	2.23	0.03*
Leadership status					
Leader	Reference				
Staff	−0.70	1.08	−2.85, 1.44	−0.65	0.52

* Indicates statistical significance.

## 4 Discussion

Informants from our study exhibited higher organizational readiness for change compared to those from other pre-implementation and implementation science studies. These studies focused on a midwifery model of care (average ORIC score = 41.5) (27), an electronic lung cancer patient outcome reporting system (average ORIC score = 47.24) (28), and a doula–hospital partnership program (average ORIC score = 49.96) (39), all within hospital settings. To our knowledge, our study is the first to examine organizational readiness for change towards implementing a sepsis survivor transition-in-care protocol from hospital to home.

The higher organizational readiness for change observed within our study may be due to (1) the already established internal workflows within the five healthcare systems and HHC agencies involved in implementing the I-TRANSFER protocol and (2) the quality and financial priorities set by Centers for Medicare and Medicaid Services (CMS) to prevent rehospitalizations (24, 40–42). Established internal workflows facilitate patient referral from hospital to post-acute care and transition-in-care protocol integration, reflecting the compatibility construct within the Consolidated Framework for Implementation Research (CFIR) (43, 44). This construct is defined as the alignment between existing workflows and the innovation to be implemented (43, 44). CMS launched initiatives, such as the Home Health Quality Reporting Program (HHQRP) and the Hospital Readiness Readmissions Reduction Program (HRRP), financially incentivizes quality care within hospitals and HHC (24, 40). This corresponds with the CFIR external pressure construct, which includes external initiatives and policies influencing implementation (43, 44). Compatibility of a transition-in-care protocol with existing workflows and external pressures may potentially increase collective capability and motivation towards implementation, leading to higher organizational readiness of change.

We compared organizational readiness of change between informants from hospitals and post-acute care (HHC and outpatient clinics) to inform sepsis survivor transition-in-care protocol implementation. Those from post-acute care had a 4.39-unit higher mean organizational readiness for change score compared to those from hospitals. Although studies have yet to compare hospital and post-acute care, potential explanations for our findings may be related to external health policies, quality measures, priorities, organizational structures, and expertise.

Health policies and quality measures set by the CMS HHQRP may influence organizational readiness for change among HHC agencies (24, 40). According to HHQRP health policies, HHC agencies must initiate a start-of-care visit within two days post-discharge. In addition, the HHQRP financially incentivizes HHC agencies to reduce 30-day rehospitalization rates (24, 40). As sepsis survivors are at high risk for 30-day rehospitalizations (9, 10), HHC informants have a strong incentive to implement sepsis survivor transition-in-care protocols aligning with these requirements, such as the I-TRANSFER protocol (20). This increases informant resolve, contributing to higher organizational readiness for change (26). Furthermore, a systematic review showed that the HHQRP contributes to HHC agency engagement in quality improvement initiatives focused on staffing, quality monitoring, and care coordination redesign, suggesting that health policies and quality measures enable HHC informants towards implementing transition-in-care protocols (40). Thus, maintaining these health policies and quality measures may be essential towards increasing organizational readiness for change.

Hospital informants may prioritize reducing 30-day rehospitalizations, but other health conditions may have a higher priority over sepsis. According to the CMS Hospital Readiness Readmissions Reduction Program (HRRP), hospitals having excess 30-day rehospitalizations for heart failure, myocardial infarction, chronic obstructive pulmonary disease, coronary artery bypass graft surgery, total knee or hip arthroplasty, and pneumonia will face reduced payments from CMS (42). As such, hospital informants may be less inclined to prioritize implementing sepsis survivor transition-in-care protocols as sepsis is not part of HRRP, resulting in lower organizational readiness for change. Given the poor outcomes among sepsis survivors (11, 45), we recommend sepsis be added to HRRP to incentivize hospitals towards implementing transition-in-care protocols tailored to sepsis survivors.

Organizational structure differences may also explain differences in organizational readiness. Although studies have yet to confirm, hospitals may have more complex organizational structures with multiple layers of committees than HHC agencies and outpatient clinics. Receiving approval from these hierarchical committees is required prior to implementing a transition-in-care protocol but may delay the implementation timeline. This delay due to bureaucratic processes may reduce implementation buy-in, motivation, and organizational readiness for change. However, the less hierarchical structure within HHC and outpatient clinics may lead to a more streamlined approach towards implementing protocols. One strategy is to organize a hospital-wide committee dedicated to improving sepsis outcomes, leading to centralized efforts towards expediting transition-in-care protocol implementation and mitigating bureaucratic barriers.

HHC agency and outpatient informants may have more expertise than hospital informants in implementing transition-in-care protocols, potentially due to differences in practice scope. Hospitals provide care to patients with diverse, acute, and complex health conditions requiring specialized services and inpatient monitoring, while HHC agencies and outpatient clinics provide mainly post-acute care, rehabilitation, and primary care to patients who transitioned from hospital care. Thus, HHC agency and outpatient informants may have expertise in monitoring and managing health conditions to prevent rehospitalizations. This expertise makes them more positioned than hospital informants towards caring out transition-in-care protocols, increasing their readiness for implementing change. Another strategy is to create interdisciplinary teams consisting of post-acute care and hospital informants to plan and execute transition-in-care protocols.

Our findings and inferences should be interpreted with caution. The study limited post-acute care to HHC and outpatient appointments. Additional studies may include additional post-acute care institutions, such as skilled nursing and rehabilitation care. While our findings are statistically significant, research is needed to establish their clinical significance. We described potential explanations for our findings, but more studies are needed to substantiate them.

Distributing surveys for data collection may have introduced selection bias, and those who completed the survey may have higher organizational readiness of change than those who did not. Due to the size of the healthcare institutions, we recruited more hospital than post-acute informants, and only four within post-acute were from outpatient clinics and the rest were from HHC. Thus, our findings are skewed towards HHC and hospitals as we did not have enough data to elucidate organizational readiness for change among outpatient informants.

Our informant sample had a larger proportion of leaders from post-acute care than from hospitals, which may have influenced responses to ORIC. Additional factors, such as clinician fatigue, time constraints, and work environments may have also influenced organizational readiness for change but were not captured within our study. Most of our informants identified as White and Non-Hispanic/Latino, limiting our findings’ generalizability to only those with similar sociodemographic characteristics or from similar HHC agencies, hospitals, and outpatient clinics.

Moving forward, future research may focus on the ORIC measure and assessing organizational readiness for change longitudinally throughout implementation. Our study's ORIC Cronbach's alpha values indicated high internal reliability, like those reported by other studies (23, 27, 46). This suggests consistent internal scale consistency across different populations. However, ORIC cut-off points for poor, fair, good, and excellent organizational readiness for change have yet to be established, and additional studies may determine the predictive validity of ORIC towards implementation success (27, 47). As implementation proceeds, informants may encounter barriers and facilitators, such as those related to time constraints, staff training, and care coordination (4). As such, researchers may consider measuring organizational readiness for change throughout implementation.

To conclude, our study lays a foundation for understanding organizational readiness for change in implementing transition-in-care protocols for sepsis survivors, but its limitations require a cautious interpretation. Present results should be considered exploratory and additional research is needed to confirm these findings, measure clinical significance, and elucidate underlying factors among larger and more diverse samples.

## 5 Conclusion

Our cross-sectional study found that informants from post-acute care had higher organizational readiness for change compared to those from hospitals for implementing sepsis survivor transition-in-care protocols. Findings may inform tailored strategies and health policies for implementing transition-in-care protocols for sepsis survivors, an at-risk population for high long-term morbidity and mortality. Strategies include creating a hospital-wide committee to improve sepsis survivor outcomes and launching interdisciplinary teams, consisting of post-acute care and hospital informants, to coordinate care for sepsis survivors transitioning from hospital to home. The CMS HHQRP should continue incentivizing HHC agencies to maintain timely HHC start-of-care visits and reduce 30-day rehospitalizations. Health policy makes may consider adding sepsis to the CMS HRRP to encourage hospitals to prioritize improving sepsis survivor outcomes.

Although our study is exploratory and its findings require a cautious interpretation, they inform effect sizes and lay the foundation for future studies on organizational readiness for change towards implementing sepsis survivor transition-in-care protocols. Future work should confirm and expand upon our findings by recruiting larger and more diverse samples, studying additional factors potentially associated with organizational readiness for change, and by investigating the predictive relationship between baseline organizational readiness for change and implementation success.

## Data Availability

The raw de-identified data supporting the conclusions of this article will be made available by the authors upon reasonable request.
