# Corrigendum: Organizational readiness for change towards implementing a sepsis survivor hospital to home transition-in-care protocol

**DOI:** 10.3389/frhs.2025.1552666

**Published:** 2025-01-24

**Authors:** Elaine Sang, Ryan Quinn, Michael A. Stawnychy, Jiyoun Song, Karen B. Hirschman, Sang Bin You, Katherine S. Pitcher, Nancy A. Hodgson, Patrik Garren, Melissa O'Connor, Sungho Oh, Kathryn H. Bowles

**Affiliations:** ^1^ NewCourtland Center for Transitions and Health, School of Nursing, University of Pennsylvania, Philadelphia, PA, United States; ^2^ Leonard Davis Institute of Health Economics, The Wharton School, University of Pennsylvania, Philadelphia, PA, United States; ^3^ Biostatistics Evaluation Collaboration Consultation Analysis (BECCA) Lab, School of Nursing, University of Pennsylvania, Philadelphia, PA, United States; ^4^ Department of Biobehavioral Health Sciences, School of Nursing, University of Pennsylvania, Philadelphia, PA, United States; ^5^ Penn Medicine Princeton Medical Center, Plainsboro Township, NJ, United States; ^6^ Gerontology Interest Group, M. Louise Fitzpatrick College of Nursing, Villanova University, Villanova, PA, United States; ^7^ Center for Home Care Policy & Research, VNS Health, New York, NY, United States

**Keywords:** sepsis survivors, transitions in care, organizational readiness for change, implementation science, healthcare system, home health care (HHC), transition-in-care protocols, hospital to home

A Corrigendum on Organizational readiness for change towards implementing a sepsis survivor hospital to home transition-in-care protocol By Sang E, Quinn R, Stawnychy MA, Song J, Hirschman KB, You SB, Pitcher KS, Hodgson NA, Garren P, O'Connor M, Oh S and Bowles KH (2024). Front. Health Serv. 4:1436375. doi: 10.3389/frhs.2024.1436375

In the published article, there was an error in Figure 1 as published. The authors mistakenly labeled Post-Acute Care as Hospital and vice versa. The corrected Figure 1 and its caption appear below.

**Figure 1:**
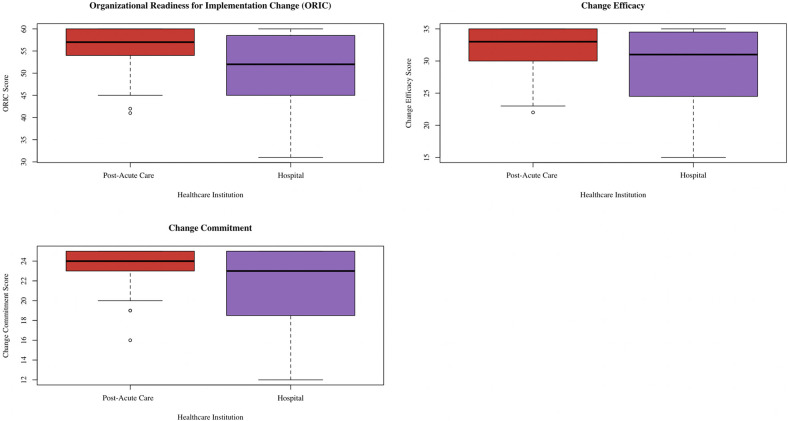
Box plot showing distribution of ORIC, change commitment, and change efficacy scores separated by healthcare institution. This box plot was created via the ggplot2 package (38) in R (37).

The authors apologize for this error and state that this does not change the scientific conclusions of the article in any way. The original article has been updated.

