# Supplementary material for: Organizational readiness for change towards implementing a sepsis survivor hospital to home transition-in-care protocol
Source: Front Health Serv. 2024 Sep 6;4:1436375. doi: 10.3389/frhs.2024.1436375 (PMC11412944; doi:10.3389/frhs.2024.1436375)
Supplement: Supplementary file 2 [file Datasheet2.docx]

**SUPPLEMENTAL FILE 2:** ORIC scale (12-items)

| **Item number** | **Subscale** | **Item Description** |
| --- | --- | --- |
| 1 | Change Efficacy | People who work here feel confident that the organization can get people invested in implementing this change. |
| 2 | Change Commitment | People who work here are committed to implementing this change. |
| 3 | Change Efficacy | People who work here feel confident that they can keep track of progress in implementing this change. |
| 4 | Change Commitment | People who work here will do whatever it takes to implement this change. |
| 5 | Change Efficacy | People who work here feel confident that the organization can support people as they adjust to this change. |
| 6 | Change Commitment | People who work here want to implement this change. |
| 7 | Change Efficacy | People who work here feel confident that they can keep the momentum going in implementing this change. |
| 8 | Change Efficacy | People who work here feel confident that they can handle the challenges that might arise in implementing this change. |
| 9 | Change Commitment | People who work here are determined to implement this change. |
| 10 | Change Efficacy | People who work here feel confident that they can coordinate tasks so that implementation goes smoothly. |
| 11 | Change Commitment | People who work here are motivated to implement this change. |
| 12 | Change Efficacy | People who work here feel confident that they can manage the politics of implementing this change. |
