# Supplementary material for: Organizational readiness for change towards implementing a sepsis survivor hospital to home transition-in-care protocol
Source: Front Health Serv. 2024 Sep 6;4:1436375. doi: 10.3389/frhs.2024.1436375 (PMC11412944; doi:10.3389/frhs.2024.1436375)
Supplement: Supplementary file 3 [file Datasheet3.docx]

**SUPPLEMENTAL FILE 3:** Characteristics of the Informant Population

| **Characteristic** | **Informant**  **Population (n=84)**  **n (%)** |
| --- | --- |
| *Gender*  Male  Female | 25 (29.76%)  59 (70.24%) |
| *Race*  White  Asian  Black  Pacific Islander  Missing | 69 (82.14%)  9 (10.71%)  1 (1.19%)  1 (1.19%)  4 (4.76%) |
| *Ethnicity*  Hispanic/ Latino  Non-Hispanic/ Non-Latino  Missing | 2 (2.38%)  78 (92.86%)  4 (4.76%) |
| *Leadership Status*  Leader  Staff | 44 (52.38%)  40 (47.62%) |
| *Healthcare Institution*  Hospital  Post-Acute Care | 51 (60.71%)  33 (39.29%) |
| **Organizational Readiness for Change** | **Mean (SD)** |
| ORIC  Change Commitment  Change Efficacy | 52.44 (8.05)  22.15 (3.45)  30.28 (4.93) |
