# Supplementary material for: Organizational readiness for change towards implementing a sepsis survivor hospital to home transition-in-care protocol
Source: Front Health Serv. 2024 Sep 6;4:1436375. doi: 10.3389/frhs.2024.1436375 (PMC11412944; doi:10.3389/frhs.2024.1436375)

**SUPPLEMENTAL FILE 4:** Frequency Bar Graph Showing Distribution of Informant Responses to All 12 Individual ORIC Items


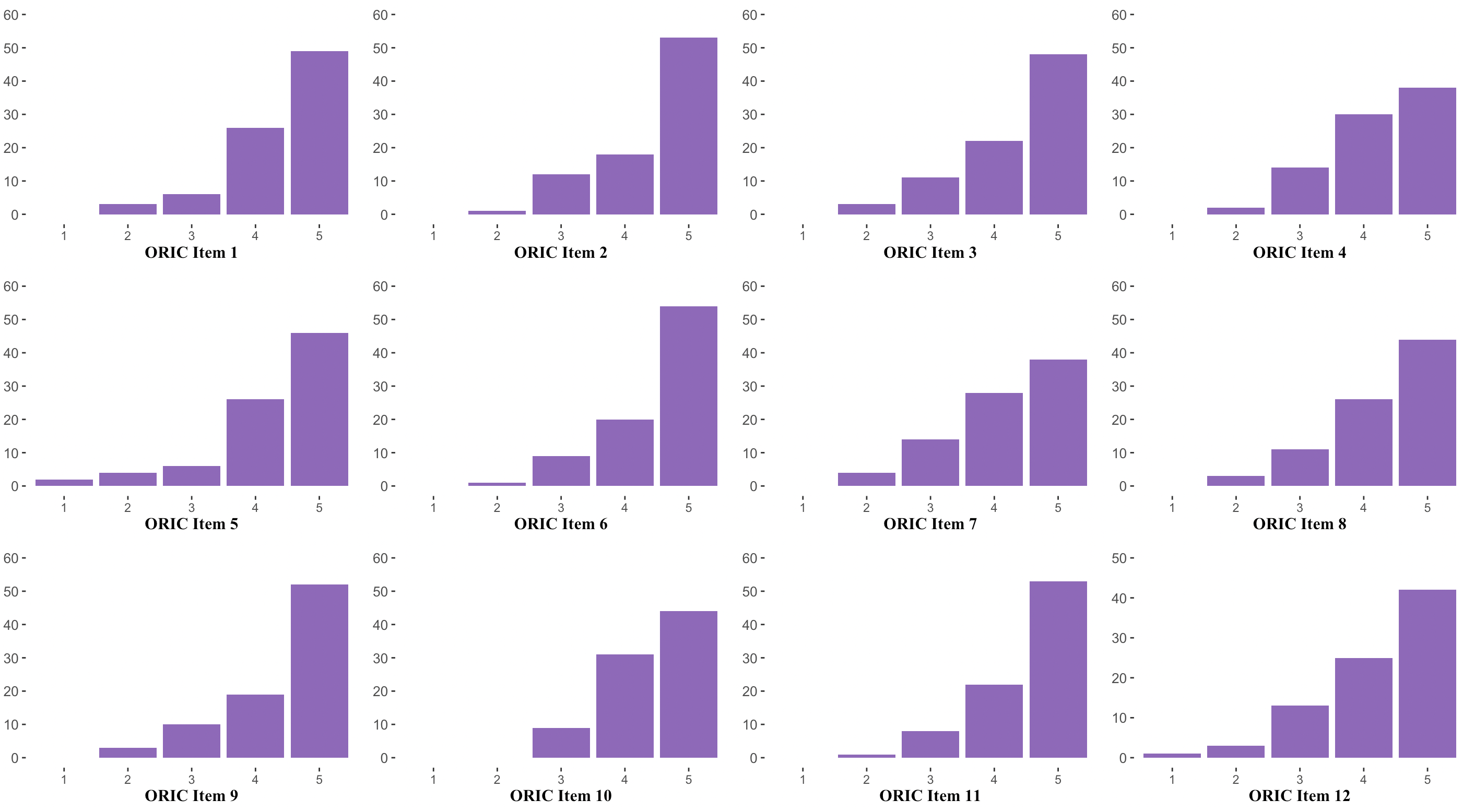

Supplement: Supplementary file 4 [file Datasheet4.docx]
