# Supplementary material for: Organizational readiness for change towards implementing a sepsis survivor hospital to home transition-in-care protocol
Source: Front Health Serv. 2024 Sep 6;4:1436375. doi: 10.3389/frhs.2024.1436375 (PMC11412944; doi:10.3389/frhs.2024.1436375)

**SUPPLEMENTAL FILE 5:** Frequency Bar Graph Showing Distribution of Informant Responses to All 12 Individual ORIC Items Separated by Healthcare Institution (Hospital and Post-Acute Care)


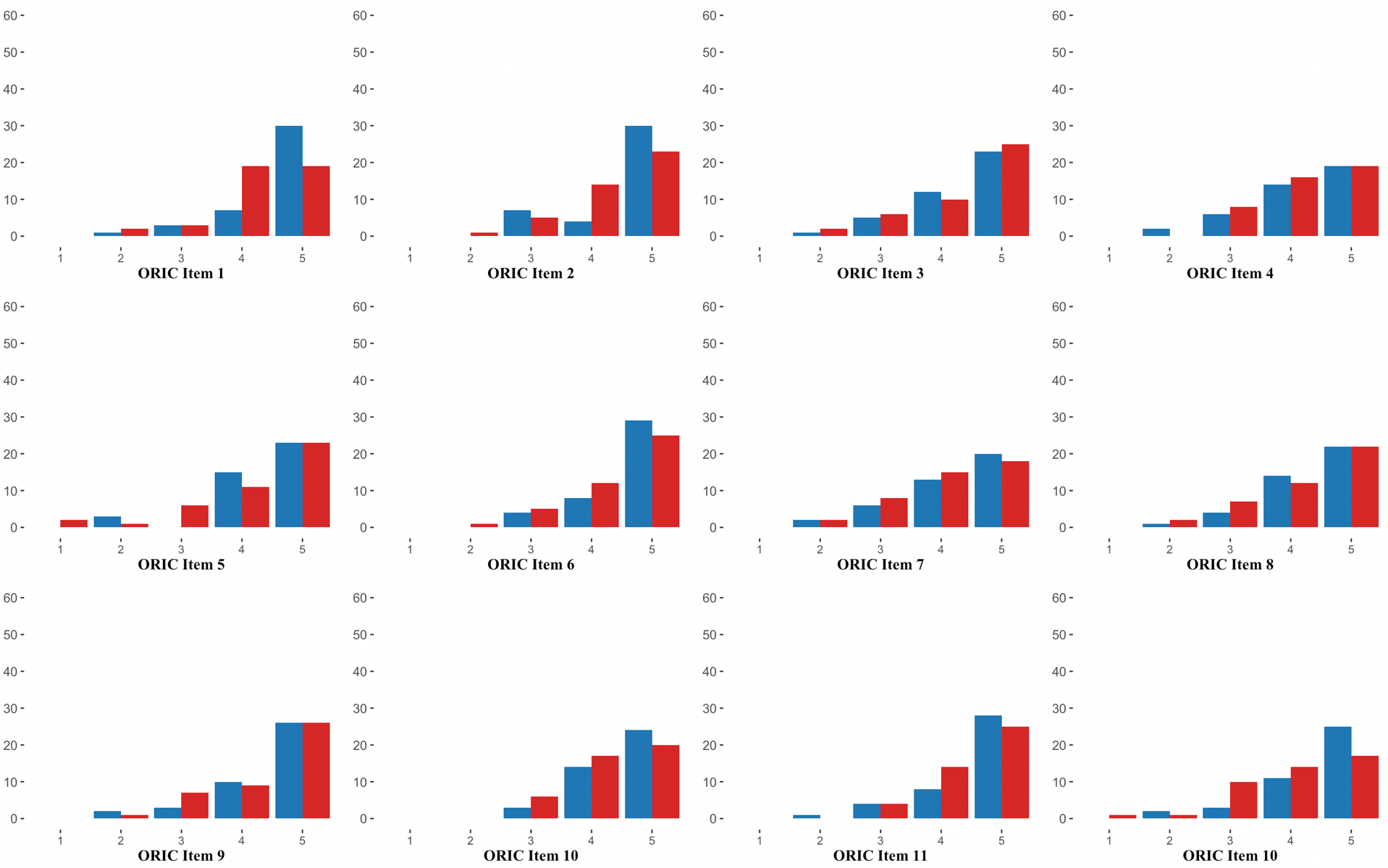


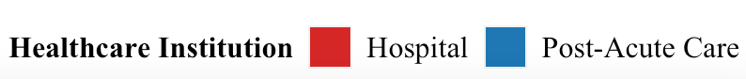

Supplement: Supplementary file 5 [file Datasheet5.docx]
