# Supplementary material for: Organizational readiness for change towards implementing a sepsis survivor hospital to home transition-in-care protocol
Source: Front Health Serv. 2024 Sep 6;4:1436375. doi: 10.3389/frhs.2024.1436375 (PMC11412944; doi:10.3389/frhs.2024.1436375)
Supplement: Supplementary file 6 [file Datasheet6.docx]

**SUPPLEMENTAL FILE 6:** Simple Linear Regression Organizational Readiness for Change Before Adjusting for Leadership vs. Non-leadership

| **Variables** | **Regression Coefficient** | **Standard Error** | **95% Confidence Interval (CI)** | ***t* value** | **P-Value** |
| --- | --- | --- | --- | --- | --- |
| **Organizational Readiness** | | | | | |
| Intercept  Healthcare Setting  Hospitals  Post-Acute Care | 50.64  Reference  4.57 | 1.09  1.74 | 48.51, 52.78  1.12, 7.69 | 46.45  2.63 | < .01*****  .01***** |
| **Change Commitment** | | | | | |
| Intercept  Healthcare Setting  Hospitals  Post-Acute Care | 21.39  Reference  1.94 | 0.47  0.75 | 20.48, 22.31  0.48, 3.40 | 45.77  2.60 | < .01*****  .01***** |
| **Change Efficacy** | | | | | |
| Intercept  Healthcare Setting  Hospitals  Post-Acute Care | 29.25  Reference  2.63 | 0.67  1.07 | 27.94, 30.57  0.53, 4.73 | 43.65  2.46 | < .01*****  .02***** |

*****Indicates statistical significance
